# Drug-induced interstitial lung disease during cancer therapies: expert opinion on diagnosis and treatment

**DOI:** 10.1016/j.esmoop.2022.100404

**Published:** 2022-02-24

**Authors:** P. Conte, P.A. Ascierto, G. Patelli, R. Danesi, A. Vanzulli, F. Sandomenico, P. Tarsia, A. Cattelan, A. Comes, M. De Laurentiis, A. Falcone, D. Regge, L. Richeldi, S. Siena

**Affiliations:** 1 DiSCOG, University of Padova and Medical Oncology 2, IOV—Istituto Oncologico Veneto IRCCS, Padua, Italy; 2 Department of Melanoma, Cancer Immunotherapy and Development Therapeutics, Istituto Nazionale Tumori IRCCS Fondazione Pascale, Naples, Italy; 3 Department of Oncology and Hemato-Oncology, Università degli Studi di Milano, Milan, Italy; 4 Department of Hematology, Oncology and Molecular Medicine, Grande Ospedale Metropolitano Niguarda, Milan, Italy; 5 Unit of Clinical Pharmacology and Pharmacogenetics, Department of Clinical and Experimental Medicine, University of Pisa, Pisa, Italy; 6 Radiology Department, Grande Ospedale Metropolitano Niguarda, Milan, Italy; 7 Radiology Unit, Buon Consiglio Fatebenefratelli Hospital, Naples, Italy; 8 Pneumology Unit, Grande Ospedale Metropolitano Niguarda, Milan, Italy; 9 Tropical and Infectious Diseases Unit, Padua University Hospital, Padua, Italy; 10 Unità Operativa Complessa di Pneumologia, Fondazione Policlinico Universitario A. Gemelli IRCCS, Università Cattolica del Sacro Cuore, Rome, Italy; 11 Department of Breast and Thoracic Oncology, Istituto Nazionale Tumori IRCCS Fondazione Pascale, Naples, Italy; 12 Unit of Medical Oncology 2, Azienda Ospedaliero-Universitaria Pisana, Pisa, Italy; 13 Department of Radiology, Candiolo Cancer Institute, FPO-IRCCS, Candiolo, Turin, Italy; 14 Department of Surgical Sciences, University of Turin, Turin, Italy

**Keywords:** antineoplastic agents, diagnostic–therapeutic algorithm, differential diagnosis, interstitial lung disease, pneumonia

## Abstract

**Background:**

Drug-induced interstitial lung disease (DIILD) is a form of interstitial lung disease resulting from exposure to drugs causing inflammation and possibly interstitial fibrosis. Antineoplastic drugs are the primary cause of DIILD, accounting for 23%-51% of cases, with bleomycin, everolimus, erlotinib, trastuzumab-deruxtecan and immune checkpoint inhibitors being the most common causative agents. DIILD can be difficult to identify and manage, and there are currently no specific guidelines on the diagnosis and treatment of DIILD caused by anticancer drugs.

**Objective:**

To develop recommendations for the diagnosis and management of DIILD in cancer patients.

**Methods:**

Based on the published literature and their clinical expertise, a multidisciplinary group of experts in Italy developed recommendations stratified by DIILD severity, based on the Common Terminology Criteria for Adverse Events.

**Results:**

The recommendations highlight the importance of multidisciplinary interaction in the diagnosis and management of DIILD. Important components of the diagnostic process are physical examination and careful patient history-taking, measurement of vital signs (particularly respiratory rate and arterial oxygen saturation), relevant laboratory tests, respiratory function testing with spirometry and diffusing capacity of the lung for carbon monoxide and computed tomography/imaging. Because the clinical and radiological signs of DIILD are often similar to those of pneumonias or interstitial lung diseases, differential diagnosis is important, including microbial and serological testing to exclude or confirm infectious causes. In most cases, management of DIILD requires the discontinuation of the antineoplastic agent and the administration of short-term steroids. Steroid tapering must be undertaken slowly to prevent reactivation of DIILD. Patients with severe and very severe (grade 3 and 4) DIILD will require hospitalisation and often need oxygen and non-invasive ventilation. Decisions about invasive ventilation should take into account the patient’s cancer prognosis.

**Conclusions:**

These recommendations provide a structured step-by-step diagnostic and therapeutic approach for each grade of suspected cancer-related DIILD.

## Highlights

• There is a lack of guidance for the management of DIILD in cancer patients.
• A multidisciplinary team in Italy developed step-by-step diagnostic and therapeutic guidelines for DIILD in cancer patients.
• Differential diagnosis of DIILD is critical to exclude other forms of interstitial lung disease or infectious causes.
• Usually the antineoplastic agent is discontinued, steroids started and further management dictated by DIILD severity.

## Introduction

Interstitial lung disease (ILD) is a collective term for a heterogeneous group of pulmonary parenchymal diseases, characterised by nonspecific clinical, radiological and pathological patterns, and occurring secondary to inflammation and fibrosis of the pulmonary interstitium.^1^^,^^2^ ILDs may be caused by several conditions, such as connective tissue disorders, environmental agents or iatrogenic events, with a diverse range of disease behaviours.^1^ Although ILDs may be triggered or exacerbated by infectious agents, these causes are not acknowledged in the current pathological classifications.^3^

Drug-induced interstitial lung disease (DIILD) defines a subset of ILDs resulting from exposure to drugs causing inflammation and possibly interstitial fibrosis.^4^ The clinical suspicion of DIILD increases as more cases are described secondary to a drug exposure, although a definitive diagnosis relies on the exclusion of other possible causes.^5^ In cancer patients, DIILD is primarily associated with cytotoxic chemotherapy, targeted therapy and immunotherapy.^4^

DIILD can be difficult to identify and manage. Most of the currently available studies on DIILD in cancer patients are retrospective and limited in sample size. Furthermore, specific guidelines on anticancer therapy-related DIILD are lacking. Currently, the main international guidelines on DIILD are not specific to cancer therapy, mainly address immunotherapy-related adverse effects and often provide inconsistent management recommendations.^6^, ^7^, ^8^ Very recently, a review article focused on the current knowledge of the pathogenesis and epidemiologic characteristics of anti-human epidermal growth factor receptor 2 (HER2) antibody-drug conjugate (ADC)-related lung toxicity, proposing strategies for its diagnosis and treatment.^9^ The authors conclude that early diagnosis and a more appropriate treatment of ADC-induced ILD may improve the therapeutic index of this relevant class of anticancer agents, allowing for a safe extension of the use of anti-HER2 ADCs across different tumour types.^9^

Diagnosis and treatment of DIILD require a specific coordinated multidisciplinary approach for optimal outcomes. Given the paucity of scientific publications and clear guidelines on DIILD in cancer patients, there is therefore a need for comprehensive guidelines on the management of such patients.

The aim of this review and guideline, developed by a multidisciplinary group of experts in Italy, is to provide health care professionals with a useful tool to identify risk factors for anticancer therapy-related DIILD, and to offer a comprehensive diagnostic–therapeutic strategy specifically for cancer patients with DIILD, based on a critical review of the literature and the authors’ clinical expertise. Some real-world case examples are also provided in the Supplementary Materials, available at https://doi.org/10.1016/j.esmoop.2022.100404.

## Methods

A multidisciplinary panel of 14 experts from across Italy carried out a targeted literature review on anticancer therapy-related DIILD to formulate diagnosis and treatment recommendations based on both scientific literature and expertise in their specialist settings: oncology, pneumology, radiology, pharmacology and infectious diseases.

The literature review was limited to English-language papers listed on the Medline (via PubMed) database and published between 1 January 2002 and 31 December 2021, and was mainly based on the following terms: ‘adverse pulmonary event’, ‘drug-induced interstitial lung pneumonia’, ‘cancer’, ‘COVID-19’, ‘immune-mediated pneumonitis’, ‘immunotherapy’, ‘immunotherapy-related pneumonia’, ‘lung toxicity’.

At the end of the first meeting, panellists were assigned to separate working groups, each including at least one representative of the five involved specialties and addressing a specific grade of DIILD. To finalise an expert recommendation, there had to be unanimous agreement amongst all panellists. All authors contributed equally to the work and share the same responsibility for the statements included in the text.

This article is based on previously conducted studies and does not contain any new studies with human participants or animals carried out by any of the authors.

## Epidemiology

DIILD accounts for 3%-5% of all ILD cases, with an incidence of 4.1-12.4 per million per year.^4^ Several drugs are potentially associated with DIILD, although in most cases only sporadically; in this regard, the Pneumotox online platform can be used as a reference for drug-related respiratory toxicities.^10^ Antineoplastic agents are acknowledged as the primary cause of DIILD (accounting for 23%-51% of all reported cases), followed by antirheumatic drugs, amiodarone and antibiotics. The risk of DIILD increases when causative drugs are used in combination and, for some drugs, can be dose-dependent.^4^

In the oncology setting, the cytotoxic agent bleomycin and mammalian target of rapamycin (mTOR) inhibitor everolimus (3%-58%) are associated with the highest incidence of DIILD (7%-21% of treated patients), followed by multiple targeted therapies [i.e. anti-epidermal growth factor receptor agents, anti-BRAF agents, cyclin-dependent kinase 4/6 inhibitors, poly (ADP-ribose) polymerase inhibitors, etc., with variable incidence] and immune checkpoint inhibitors (ICIs; 1%-4%).^4^^,^^11^, ^12^, ^13^, ^14^ Case-fatality rates vary between 0% and 51.3% according to different drugs.^4^

Among novel drugs, the Food and Drug Administration and European Medicines Agency-approved HER2-targeting ADC trastuzumab-deruxtecan (T-DXd) carries a known risk of DIILD with 15.8% incidence (mostly low grade) and 2.4% mortality in clinical trials.^15^, ^16^, ^17^ However, in the latest phase III DESTINY-Breast03, no T-DXd-related deaths and very severe forms (grade 4) occurred and DIILD incidence was 10.5%.^18^

The main classes of anticancer agents causing DIILD and its incidence are shown in Supplementary Table S1, available at https://doi.org/10.1016/j.esmoop.2022.100404.

## Pathogenesis

Pathogenic mechanisms of DIILD are not yet completely understood. A commonly accepted hypothesis is that the causative drug exerts an immunological effect by direct haptenic modification of tissue-resident proteins or by antibody–antigen immune complex deposition, followed by inflammatory response.^19^^,^^20^ Another proposed mechanism is direct toxic effects on endothelial and epithelial cells; this mechanism has been observed in patients treated with bleomycin, amiodarone or phenytoin, who present with significant neutrophilia, and in patients treated with methotrexate or nitrofurantoin, who develop lymphocytic alveolitis.^19^^,^^20^ Drugs such as cyclophosphamide, amiodarone, carmustine, nitrofurantoin and bleomycin are metabolised in the lungs and may induce the release of highly cytotoxic reactive oxygen species leading to pulmonary injuries; some other agents can increase endothelial permeability; others, such as the phospholipase A2 inhibitor amiodarone, can cause phospholipid accumulation within the alveolar cells, resulting in degenerative and regressive alterations in lung macrophages and alveolar cells, or can interact with other pharmacological agents, or cause the release of cytokines and chemokines resulting in inflammatory response.^19^^,^^20^

Possible mechanisms of toxicity caused by T-DXd could be a target-dependent uptake of ADC and/or target-independent uptake and catabolism of ADC in normal cells, or ‘bystander effect’ by the cytotoxic payload released from cells following catabolism of the ADC.^21^, ^22^, ^23^ Interestingly, deconjugated deruxtecan did not cause DIILD in animal models and the distribution of HER2-tissue expression (low level of expression in respiratory alveoli) failed to corroborate the target-dependent uptake hypothesis, likely leaving target-independent uptake of the conjugate by immune cells as the main pathogenic explanation.^9^ DIILD may develop from days to months after drug administration, so late clinical manifestations do not exclude the possibility of DIILD.^4^ However, the majority of ILD events are reported to occur early in the course of treatment, within the first 2 months for ICIs and in the initial 12 months with T-DXd.^8^ With the latter agent, the risk of late onset is reduced to 7.0% after 12 months and 1.4% after 18 months.^17^

## Risk factors

Before the start of any anticancer therapy, physicians should carefully evaluate the risk for DIILD (Supplementary Table S2, available at https://doi.org/10.1016/j.esmoop.2022.100404),^4^ although risk factors vary among studies (which have mainly been retrospective) and anticancer drugs.^4^^,^^24^^,^^25^

During history-taking, it is essential to obtain information on any non-cancer-related concomitant medications that may potentially cause DIILD (Supplementary Table S3, available at https://doi.org/10.1016/j.esmoop.2022.100404).^4^ Asian ethnicity may represent an important hazard for T-DXd-related DIILD according to a pool analysis of the DESTINY trials.^26^ Besides, previous manifestation of DIILD is reported among relevant risk factors for recurrence upon drug rechallenge, with a 25%-30% absolute risk for ICIs.^4^^,^^27^ However, given the peril of such drug reintroduction, data mostly come from case reports and retrospective analyses with small sample size.

## Classifying severity

DIILD can present with a spectrum of clinical severities depending on the extent of involvement of the lung interstitium and the patient’s clinical condition, and therefore can vary in clinical manifestations and outcomes even with the same agent. In oncology, DIILD severity is graded according to clinical manifestations, in accordance with the Common Terminology Criteria for Adverse Events (CTCAE v5.0) (Supplementary Table S4, available at https://doi.org/10.1016/j.esmoop.2022.100404).^28^

## Radiological and pathological features

Radiological diagnosis is essential for appropriate management. The imaging technique of choice is computed tomography (CT) of the chest, particularly high-resolution CT (HRCT), for its high sensitivity and specificity, and its capacity to grade the extent of lung involvement. Up to one-third of patients with DIILD can be asymptomatic, so incidental diagnosis in patients with radiological evidence of interstitial pneumonia may occur.^29^

The main CT patterns of pneumonia are: acute interstitial pneumonia (AIP) (Supplementary Figure S1A, available at https://doi.org/10.1016/j.esmoop.2022.100404), organising pneumonia (OP) (Supplementary Figure S1B, available at https://doi.org/10.1016/j.esmoop.2022.100404), nonspecific interstitial pneumonia (NSIP) (Supplementary Figure S1C, available at https://doi.org/10.1016/j.esmoop.2022.100404), hypersensitivity pneumonia (HP) (Supplementary Figure S1D, available at https://doi.org/10.1016/j.esmoop.2022.100404) and acute respiratory distress syndrome (ARDS).^30^^,^^31^

AIP is characterised by thickening of the alveolar walls, deposition of hyaline membranes and infiltration of inflammatory cells; CT features are areas of ground-glass opacity (GGO), consolidation and lung volume reduction.^2^^,^^32^^,^^33^

OP results from the proliferation of granulation tissue in the lumina of distal bronchioles and alveoli. Histological findings include agglomerates of collagen-rich granulation tissue, fibroblasts and myofibroblasts in distal airspaces with infiltrate of lymphocytes and plasma cells. CT features are multifocal areas of GGO and consolidation with a predominantly peripheral distribution. Reversed halo signs, or atoll signs, with central ground-glass hyperattenuation areas surrounded by ring-shaped air space consolidation were reported.^34^

NSIP is characterised by fibrosis, infiltration of diffuse inflammatory cells and homogeneous and diffuse thickening of the alveolar walls, without loss of alveolar structural integrity. CT findings include GGO and prevalent basal and peripheral reticular opacities.^35^

Features of HP are granulomas, mainly centrilobular, chronic interstitial lymphocytic inflammation, interstitial fibrosis and alveolar inflammation. Tissue biopsy can reveal noncaseating granulomas. CT findings include diffuse GGO, centrilobular nodules and air trapping.^36^

Interstitial lung abnormalities (ILAs) are frequently observed in older patients, particularly smokers. ILAs are defined as the incidental identification of abnormalities on HRCT scans carried out without clinical suspicion of ILD (in the case of DIILD, ILAs should be interpreted as grade 1).^37^ On CT, they affect at least 5% of any lung zone and appear as GGO or reticular opacities, traction bronchiectasis, honeycombing and cysts. They can be classified into non-subpleural, subpleural without evidence of fibrosis and subpleural with evidence of fibrosis.^37^ In some cases, ILAs can be an early manifestation of an underlying disease or are associated with a risk of progression of subclinical abnormalities in the context of an already known disease.^37^ This is particularly the case in the fibrotic ILA subtype with predominantly subpleural localisation, which is frequently associated with a higher mortality risk compared with other subtypes.^37^ A significant proportion (73%) of ILAs show imaging progression to ILD.^38^

## Prevention

As suggested by the progressive decrease in incidence and severity of T-DXd-related DIILD in the latest DESTINY trials, an association between improved prognosis and raising of physicians’ awareness of DIILD may be inferred.^15^^,^^16^^,^^18^ In support of the detrimental effect of delayed DIILD diagnosis, the onset of pneumonitis was retrospectively found to be commonly earlier than that reported by the investigators in the first DESTINY trials.^39^ On the contrary, an early identification of DIILD could favour better outcomes through the application of timely and effective treatment. Besides, in case of drugs at high risk for DIILD, we recommend: (i) a thorough evaluation of individual risk factors; (ii) the baseline evaluation of respiratory function (i.e. spirometry) in addition to vital signs, physical examination and chest imaging; (iii) the adoption of diagnostic and therapeutic algorithm (as the one provided herein); (iv) the establishment of a fast-track network of multidisciplinary experts for prompt consultation; (v) an adequate communication to patients about the risks of DIILD and its clinical manifestations, aiming at early physician consultation in case of new-onset symptoms.

## Diagnostic approach

### Clinical examination

The aims of the history and clinical examination are to obtain detailed information on the drugs taken by the patient, comorbidities and any potential risk factors, as previously listed. It is also important to rule out any other cause of ILD (e.g. infections, cardiopathy, radiotherapy, progression of an underlying ILD or lung condition) and to define the temporal relationship between the onset of symptoms and exposure to the potentially causative drug.

DIILD symptoms are generally nonspecific, with the most frequent being non-productive cough, asthenia and chest pain. Dyspnoea, low-grade fever, cough, fatigue, chest pain and tightness should be carefully evaluated. Dyspnoea on exertion, when present, is the most important symptom to monitor because worsening dyspnoea could exacerbate the patient’s clinical course. Cough is most often non-productive and, except in the case of diffuse alveolar haemorrhage, is rarely associated with haemoptysis.^40^

Chest examination may detect alterations in the normal vesicular murmur and typical pulmonary crackles. In addition, patients should be examined for systemic signs, such as mucocutaneous cyanosis if hypoxia is present, or skin rashes and adenopathies that may indicate an infection.^41^

### Vital signs

When DIILD is suspected, vital signs should be routinely monitored, particularly the respiratory rate to detect tachypnoea and oxygen saturation by pulse oximetry (SpO_2_), the latter being extremely relevant in patients with dyspnoea. Abnormal SpO_2_ levels should be verified by arterial blood gas analysis. Acute respiratory failure is defined by an arterial partial pressure of oxygen (PaO_2_) below 60 mmHg; however, a comparison with basal values, if available, is always decisive, as DIILD can manifest with reduced PaO_2_ levels that are still within normal limits.^42^

### Laboratory testing

A blood sample is recommended for a complete blood count with differential, and tests for liver and kidney function and inflammatory markers, such as erythrocyte sedimentation rate, C-reactive protein and lactate dehydrogenase. In the case of circulatory shock, a procalcitonin assay should also be carried out.^43^

There are promising developments in the use of serum biomarkers for ILDs. Krebs von den Lungen-6 (KL-6) is a glycoprotein expressed by type II pneumocytes and bronchial epithelial cells in response to cellular damage and tissue regeneration. A recent study showed an increase in circulating KL-6 levels in DIILD patients that correlated with patterns of diffuse alveolar damage (DAD) and the extent of the lung injury.^44^ However, further studies are needed to define the role of biomarkers during DIILD diagnosis and follow-up.

### Microbial and serological tests

Microbial and serological tests are not specific for DIILD, but help to rule out other possible aetiologies (see Differential Diagnosis below). The most common infectious causes of interstitial pneumonia are viruses [AH1N1 influenza virus, severe acute respiratory syndrome coronavirus 2 (SARS-CoV-2), respiratory syncytial virus, cytomegalovirus, Epstein–Barr virus, adenovirus, metapneumovirus], bacteria (*Haemophilus*, *Streptococcus*, *Pseudomonas* spp., *Moraxella catarrhalis*, *Mycoplasma* and *Chlamydophila pneumoniae*, *Legionella pneumophila*, *Mycobacterium tuberculosis*) and fungi (*Aspergillus fumigatus*, *Pneumocystis jirovecii* and, in exceptional/anecdotal cases, *Candida* spp.).^3^ Several biological samples may be collected, taking into account the clinical presentation, patient history and risk factors, including samples of blood, urine, nasopharyngeal swab specimens, bronchoalveolar lavage (BAL) and protected specimen brushes, to avoid contamination from upper airway flora.^45^ Clinicians should be aware of the limitations, sensitivity and specificity for each test. For example, it is necessary to know how to distinguish a true infection from a contamination/colonisation.^46^ Moreover, some serological tests (such as those for *Mycoplasma* and *Chlamydophila* spp.) may cross-react, producing false-positive results.^47^ Therefore, results of every microbiological test should always be critically evaluated. This is crucial to avoid overtreatment, inappropriate antibiotic use and possible toxicities. Recently, new diagnostic tools like culture-independent metagenomic analysis (such as detection of 16S ribosomal RNA bacterial genes) and polymerase chain reaction (PCR) techniques have become available for the detection of a broad spectrum of viral, bacterial, fungal and protozoal agents in different body fluids (blood, respiratory samples, cerebrospinal fluid).^48^^,^^49^ Assays that detect fungal antigens, such as (1-3)-β-d-glucan and galactomannan, are useful in the diagnosis of invasive fungal infections.^50^ Assessment of galactomannan levels in BAL may also be carried out for the diagnosis of aspergillosis. Although expensive, these innovative techniques provide results much more quickly than standard cultures, facilitating the timely start of appropriate treatment and thereby potentially improving clinical outcomes for patients. When infection is suspected, we recommend consulting an infectious disease specialist. An empiric antibiotic course may be started according to the clinical risk.^41^^,^^51^

### Respiratory function tests

The medical examination should be followed by respiratory function tests. Spirometry and diffusing capacity of the lung for carbon monoxide (DLCO) are valid tools for the evaluation of patients with suspected DIILD. Spirometry/DLCO is an easy and non-invasive test for follow-up and is rapidly available as needed if respiratory symptoms occur. A baseline assessment with these tests should be carried out as soon as DIILD is suspected, and repeated over time to monitor respiratory function. DIILD, like other ILDs, shows a restrictive spirometric pattern with a decline in total lung capacity. Some studies have shown that a decline in forced vital capacity is associated with disease progression.^52^^,^^53^ Whereas spirometry lacks the specificity for an accurate diagnosis, a reduction in DLCO is the most sensitive indicator for interstitial involvement, suggesting a worse prognostic outcome.^54^ Pulmonary function tests are contraindicated during the acute phase of grade ≥3 DIILD (i.e. during respiratory failure) and should preferably be postponed until improvement. However, an evaluation by a pneumologist is always recommended for the diagnostic work-up and follow-up.^41^

### Bronchoscopy, BAL and biopsy

Bronchoscopy and BAL, if clinically feasible, are useful diagnostic tools. BAL should be considered when there is a lack of clinical improvement on withdrawal of the causative drug, despite corticosteroid therapy, or when the differential diagnosis is inconclusive.^55^ Indeed, BAL increases the sensitivity of microbiological investigations when ruling out an infectious aetiology. Furthermore, it can allow a preliminary cytological evaluation to define the pathological histotype (e.g. CD8+ lymphocytosis is suggestive of fibrosing ILD). An abnormal cell count on BAL is not specific for DIILD since an increase in lymphocytes, neutrophils or eosinophils is also found in other pneumonias. Rather, BAL is generally used to exclude infectious pneumonia, alveolar haemorrhage and metastatic/lymphangitic tumour spread.^56^

Lung biopsy is suggested when the above-described investigations result in an uncertain diagnosis or to rule out pneumonia of any other origin, even if it is rarely carried out in advanced stage cancer patients.^41^ A biopsy, either during bronchoscopy or surgery (preferably video-assisted thoracoscopic surgery), can reveal characteristic histological features, such as NSIP, HP, OP and DAD.

### Radiological evaluation

HRCT is recommended immediately after the medical examination. HRCT is currently the most sensitive diagnostic modality for detecting ILD since its early stages. A follow-up CT scan should be repeated 2 weeks after the initial diagnosis of DIILD, although the time interval can be adjusted depending on the patient’s overall clinical course. A two-view chest X-ray may also be considered for inpatient follow-up and assessment of therapeutic response.^25^^,^^41^

## Differential diagnosis

Differential diagnosis of DIILD includes various pathologies such as infectious pneumonia, radiation pneumonitis, diffuse alveolar haemorrhage, pulmonary oedema and, more rarely, lymphangitic carcinomatosis.

There is considerable overlap in the clinical, histopathological and radiological features of interstitial pneumonias and secondary conditions, and of infectious and non-infectious forms of such pneumonias.^3^

The main infectious agents implicated in the aetiopathogenesis of ILD are shown in Supplementary Table S5, available at https://doi.org/10.1016/j.esmoop.2022.100404. Infectious causes should always be considered in patients with ILD, particularly if they are immunocompromised, have comorbidities, need blood transfusions or are receiving multiple treatments, because these patients are at increased risk of infections. Specific risk factors for an infectious origin include a previous lung transplantation, cortisone treatment and recent travel to infectious pneumonia-endemic areas.

The confirmation or exclusion of an infectious cause is therefore essential during diagnosis of DIILD, and requires a microbial and sputum culture, a QuantiFERON blood test, serologic testing, a PCR of BAL fluid and radiological exams. Lung biopsy for histopathological study should be restricted to highly selected cases due to the potentially unfavourable risk–benefit ratio in cancer patients. The diagnostic work-up requires a multidisciplinary approach involving infectious disease specialists, radiologists, oncologists and, when possible, pathologists.

DIILD needs to be considered in the differential diagnosis of bacterial pneumonia, influenza A and B viral pneumonia, actinic pneumonia and coronavirus disease 19 (COVID-19) viral pneumonia.

### Bacterial pneumonia

Typical clinical manifestations of bacterial pneumonia are asymmetrical consolidations, contextual air bronchogram and pleural effusion (Supplementary Figure S2A, available at https://doi.org/10.1016/j.esmoop.2022.100404). However, the lack of improvement with antibiotics and negative cultures (sputum, BAL and pleural fluid) may support a diagnosis of DIILD.^57^

### Influenza A, B-related pneumonia

The main CT features of influenza pneumonia are areas of GGO with parenchymal consolidation (63%), nodules (71%), linear opacities (71%), thickening of the interlobular septa and tree-in-bud signs.^58^ A differential diagnosis with viral pneumonia is considerably more difficult (Supplementary Figure S2B, available at https://doi.org/10.1016/j.esmoop.2022.100404) and is grounded on the finding of the causative agent in nasopharyngeal swabs.

### COVID-19-related pneumonia

The main CT features of COVID 19-related pneumonia are multifocal areas of GGO (96.8%), predominantly bilateral and peripheral (Supplementary Figure S2C, available at https://doi.org/10.1016/j.esmoop.2022.100404). Additional significant CT features of COVID-19 infection are crazy-paving pattern (Supplementary Figure S2D, available at https://doi.org/10.1016/j.esmoop.2022.100404) (seen in 75.4% of patients), interlobular septal thickening (37.3% of patients), air bronchogram (39.7% of patients) and reversed halo sign (23.8% of patients).^51^ As with any viral pneumonia, a nasopharyngeal swab is essential for differential diagnosis with DIILD.

### Invasive aspergillosis, fungi and other rare aetiologies

Invasive aspergillosis can be suspected when parenchymal opacities are surrounded by GGOs (‘halo’ sign), but radiologic aspects are never pathognomonic and infectious tests should always be carried out. Patients with aspergillosis are usually neutropenic or receiving corticosteroids.^3^^,^^50^ Similarly, *P. jirovecii* can cause nonspecific GGOs, reticular opacities or septal thickening, and should be excluded in patients with cancer (especially those immunocompromised).^30^ Other fungal infections (i.e. histoplasmosis) should also be considered in case of non-resolving pneumonitis in endemic areas. Finally, other rare aetiologies such as nontuberculous mycobacteria, *Nocardia* and *Actinomyces* might necessitate further investigations in particular situations.^3^

### Radiation pneumonitis

Radiation pneumonitis usually involves a section of pulmonary parenchyma that has been exposed to radiation exceeding 30-40 Gy and is not delimited by anatomical borders such as interlobar fissures and bronchovascular structures. The onset of radiation pneumonitis occurs about 6-10 weeks after radiotherapy. Typical CT features are GGOs which may increase in density and consolidate over time (Supplementary Figure S2E and F, available at https://doi.org/10.1016/j.esmoop.2022.100404).^59^

### Volume overload

Volume overload causes changes at HRCT that can be difficult to differentiate from DIILD. These changes are: areas of GGO, interlobular septal thickening, peribronchovascular interstitial thickening, increased vascular calibre and pleural effusion or thickening of fissures.^60^

**Figure 1:**
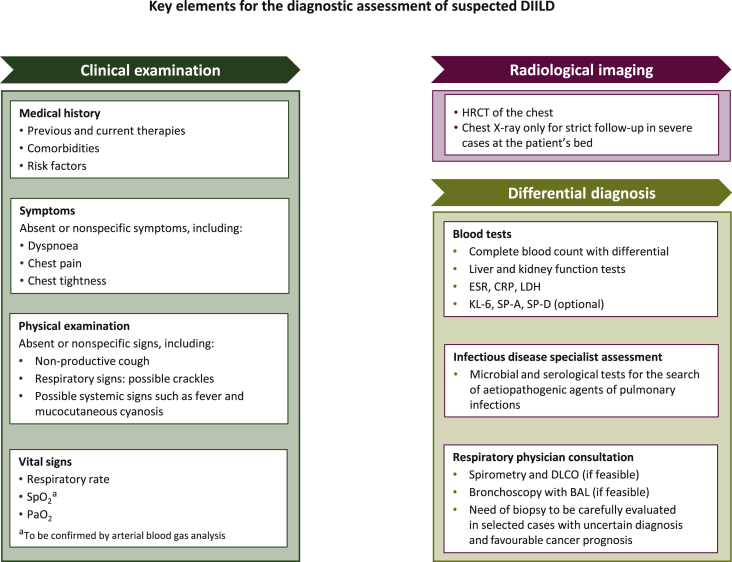
**Key elements for the baseline assessment of suspected DIILD in cancer patients.** BAL, bronchoalveolar lavage; CRP, C-reactive protein; DIILD, drug-induced interstitial lung disease; DLCO, diffusing capacity of lung for carbon monoxide; ESR, erythrocyte sedimentation rate; HRCT, high-resolution computed tomography; KL-6, Krebs von den Lungen-6; LDH, lactate dehydrogenase; PaO_2_, arterial partial pressure of oxygen; SP, surfactant protein; SpO_2_, oxygen saturation by pulse oximetry.

A list of the essential elements for the baseline assessment of DIILD in cancer patients receiving targeted therapy or immunotherapy is shown in Figure 1. Specific recommendations for CTCAE grade 1-4 DIILD are described below.

## Treatment and follow-up

Treatment approach in case of DIILD mainly consists in the discontinuation of the offending drug and start of immunosuppressive therapy, and is always driven by the grade of severity of the clinical manifestations. Since early treatment of DIILD is critical for improved outcome, a definitive diagnosis (by means of exclusion of all the alternative aetiologies) is not always mandatory before the start of steroid treatment, especially in severe cases (grade 3 and 4).

### Grade 1 DIILD

**Figure 2:**
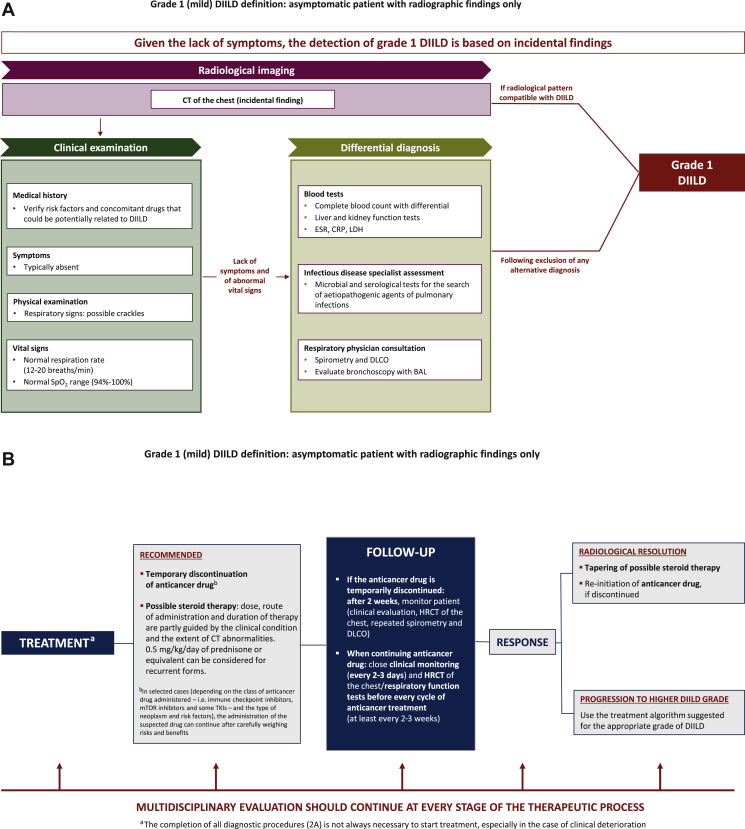
(A) Diagnostic algorithm and (B) algorithm for treatment and follow-up of grade 1 DIILD in cancer patients. Grade 1 DIILD definition is adapted from the Common Terminology Criteria for Adverse Events (CTCAE), Version 5.0 (27 November 2017).^28^ BAL, bronchoalveolar lavage; CRP, C-reactive protein; CT, computed tomography; DIILD, drug-induced interstitial lung disease; DLCO, diffusing capacity of lung for carbon monoxide; ESR, erythrocyte sedimentation rate; HRCT, high-resolution computed tomography; LDH, lactate dehydrogenase; mTOR, mammalian target of rapamycin; SpO_2_, oxygen saturation by pulse oximetry; TKIs, tyrosine kinase inhibitors.

Grade 1 (mild) DIILD is defined by the absence of respiratory symptoms, with radiographic findings only.^28^ Figure 2A shows a diagnostic algorithm, and Figure 2B a therapeutic algorithm, for grade 1 DIILD in cancer patients receiving targeted therapy or immunotherapy.

In grade 1 DIILD, it is advisable to discontinue the causative drug and to monitor the patient’s clinical condition until radiological resolution. In selected cases, the suspected drug can be continued in the presence of persistent grade 1 toxicity, but patients must be closely followed up in order to promptly intervene if their condition worsens. The decision to continue therapy requires a careful weighing of the risks and benefits, taking into account the class of anticancer drug administered (such as ICIs, ADCs and tyrosine kinase inhibitors), the type of neoplasm and the patient’s risk factors. Specifically, continuation can be considered for ICIs and mTOR inhibitors (and also tyrosine kinase inhibitors by our expert opinion), whereas ADCs such as T-DXd should be discontinued until resolution.^6^^,^^8^^,^^61^^,^^62^ Upon DIILD resolution, T-DXd can be reintroduced at the same dosage or reduced by one dose level depending on the time-span between onset and rechallenge (same dose if ≤28 days, lower dose level if >28 days).^61^

The efficacy of corticosteroids is not clearly established, given the widespread practice of discontinuing the pneumotoxic drug when starting steroid therapy. Steroid therapy is not generally needed for grade 1 DIILD; moreover, steroids may have a negative impact on survival if the patient is treated with ICIs.^6^^,^^8^^,^^63^ If steroid therapy is deemed appropriate (as in the case of T-DXd DIILD or in recurrent forms of grade 1 DIILD), the dose, route of administration and duration of steroid therapy are partly guided by the patient’s clinical condition and the extent of CT abnormalities. In these cases, the administration of 0.5 mg/kg/day of prednisone or equivalent should be considered until improvement, followed by gradual tapering over at least 4 weeks.

The prognosis for grade 1 DIILD is generally favourable. The risk of interstitial pneumonia should be assessed on a case-by-case basis, carefully weighing the risks and benefits of discontinuing a potentially effective anticancer drug and the potential clinical worsening when anticancer treatment is continued. To date, no robust scientific evidence is available to guide this decision. Generally, a follow-up visit with chest CT scan, spirometry and DLCO is acceptable after 2 weeks if the patient remains asymptomatic, whereas closer clinical monitoring (in 2-3 days) and serial imaging/respiratory function tests are recommended if the anticancer drug is continued (ideally before every cycle of treatment).

An illustrative grade 1 DIILD case study is reported in the Supplementary Materials, available at https://doi.org/10.1016/j.esmoop.2022.100404.

### Grade 2 DIILD

**Figure 3:**
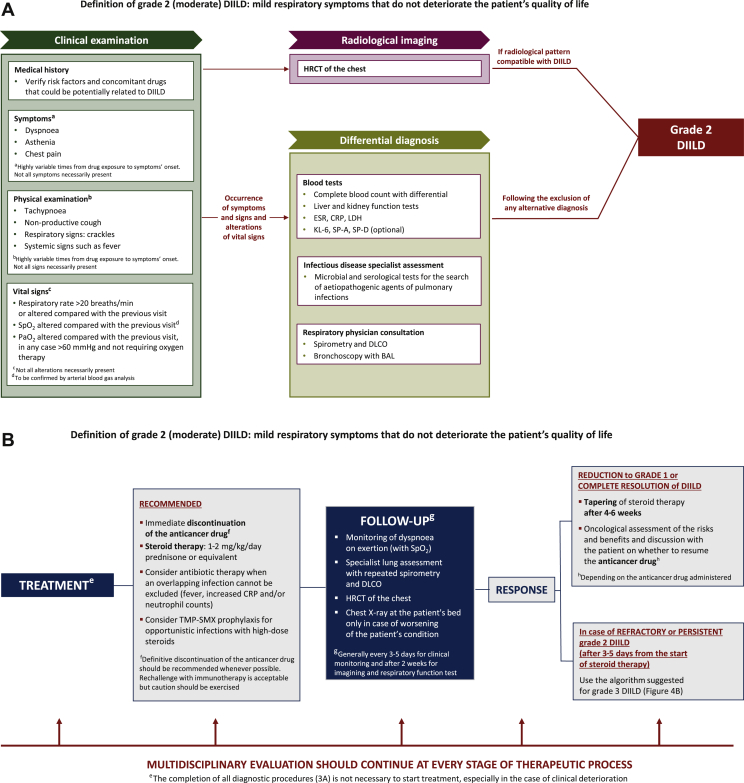
(A) Diagnostic algorithm and (B) algorithm for treatment and follow-up of grade 2 DIILD in cancer patients. Grade 2 DIILD definition is adapted from the Common Terminology Criteria for Adverse Events (CTCAE), Version 5.0 (27 November 2017).^28^ BAL, bronchoalveolar lavage; CRP, C-reactive protein; DIILD, drug-induced interstitial lung disease; DLCO, diffusing capacity of lung for carbon monoxide; ESR, erythrocyte sedimentation rate; HRCT, high-resolution computed tomography; KL-6, Krebs von den Lungen-6; LDH, lactate dehydrogenase; PaO_2_, arterial partial pressure of oxygen; SP, surfactant protein; SpO_2_, oxygen saturation by pulse oximetry; TMP-SMX, trimethoprim/sulfamethoxazole.

Grade 2 (moderate) DIILD is defined by the onset of mild respiratory symptoms that do not negatively impact the patient’s quality of life.^28^ Diagnostic and treatment algorithms for grade 2 DIILD in cancer patients receiving targeted therapy or immunotherapy are shown in Figure 3A and B, respectively.

In grade 2 DIILD, prompt discontinuation of the anticancer drug is essential, as is the initiation of corticosteroid therapy with prednisone (or equivalent) at 1-2 mg/kg/day, as recommended by the recently updated guidelines from the Society for Immunotherapy of Cancer.^6^ In patients who have been receiving T-DXd, ≥1 mg/kg/day of prednisolone (or equivalent) should be promptly administered and continued for at least 14 days.^61^ Steroid therapy can be tapered over a course of 4-6 weeks in patients who show a good response to treatment with complete resolution of hypoxia, but rapid tapering increases the risk of reactivating DIILD or worsening of the existing DIILD. Steroids should be discontinued at least 6 weeks after administration of the first dose in patients whose DIILD was caused by specific anticancer drugs, such as T-DXd.^61^ In most cases, the anticancer therapy should be permanently discontinued, but patients with a particularly favourable response may be able to restart the anticancer treatment after oncological assessment of the risks and benefits and discussion of these with the patient. In particular, drug rechallenge should be considered after grade 2 DIILD if: (i) the patient previously experienced remarkable clinical benefit from the offending drug (i.e. partial/complete response or prolonged stability of cancer disease); (ii) there was a complete resolution of the clinical and radiological abnormalities of DIILD; (iii) there are limited risk factors for DIILD recurrence and worsening (i.e. respiratory comorbidities, limited lung involvement by the tumour); (iv) in the case of ICIs, mTOR inhibitors and most targeted agents, but not T-DXd.^6^^,^^8^^,^^61^^,^^62^ Dose reduction may be deemed necessary for some anticancer agents such as everolimus (from 10 to 5 mg/day), hence thorough consultation of drug data sheet is recommended before the rechallenge (Supplementary Table S6, available at https://doi.org/10.1016/j.esmoop.2022.100404).^62^ If grade 2 DIILD is refractory or persistent after 3-5 days of steroid therapy, refer to the suggested treatment options for grade 3 DIILD.

The prognosis of grade 2 DIILD depends primarily on the response to steroid therapy, the evolution of the clinical condition (to be monitored by clinical evaluation every 3-5 days, serial imaging and spirometric investigations every 2 weeks until resolution), the extent and type of the radiological picture (in particular, pulmonary fibrosis is indicative of unfavourable prognosis) and the severity of lung function impairment. The risk factors for a worse prognosis include a history of smoking, use of drugs that are associated with high DIILD-related mortality, the patient’s age and comorbidities.^41^

An illustrative grade 2 DIILD case study is reported in the Supplementary Materials, available at https://doi.org/10.1016/j.esmoop.2022.100404.

### Grade 3 DIILD

**Figure 4:**
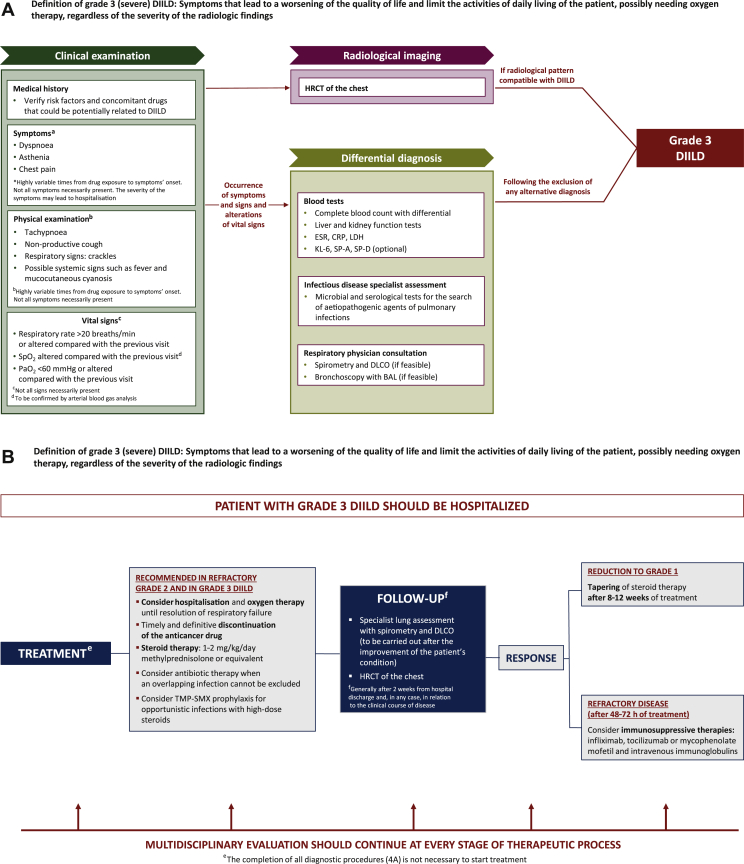
(A) Diagnostic algorithm and (B) algorithm for treatment and follow-up of grade 3 DIILD in cancer patients. Grade 3 DIILD definition is adapted from the Common Terminology Criteria for Adverse Events (CTCAE), Version 5.0 (27 November 2017).^28^ BAL, bronchoalveolar lavage; CRP, C-reactive protein; DIILD, drug-induced interstitial lung disease; DLCO, diffusing capacity of lung for carbon monoxide; ESR, erythrocyte sedimentation rate; HRCT, high-resolution computed tomography; KL-6, Krebs von den Lungen-6; LDH, lactate dehydrogenase; PaO_2_, arterial partial pressure of oxygen; SP, surfactant protein; SpO_2_, oxygen saturation by pulse oximetry; TMP-SMX, trimethoprim/sulfamethoxazole.

Grade 3 (severe) DIILD is defined by the occurrence of symptoms that lead to a worsening of the patient’s quality of life and limit their activities of daily living, including the possible need for oxygen therapy, regardless of the radiological severity.^28^ A diagnostic algorithm for grade 3 DIILD in cancer patients is shown in Figure 4A. Severe symptoms may lead to hospitalisation, as shown in the therapeutic algorithm (Figure 4B).

Hypoxic patients should receive oxygen therapy according to the degree of hypoxemia until resolution of the respiratory failure. In grade 3 DIILD, the timely and definitive discontinuation of the anticancer drug and the initiation of corticosteroid therapy at 1-2 mg/kg/day of methylprednisolone or equivalent are essential. Patients who have been receiving T-DXd should be promptly treated with ≥1 mg/kg/day of prednisolone (or equivalent).^61^ In patients who have a good response, with DIILD reverting to grade 1 (complete resolution of the symptoms with possible persistence of the radiological features), steroid therapy can be progressively tapered after 8-12 weeks; rapid steroid de-escalation increases the risk of DIILD reactivation. In patients who are refractory to steroids (no improvement within 48-72 h of starting steroids), treatment with infliximab, tocilizumab or mycophenolate mofetil and immunomodulating agents (intravenous immunoglobulins) may be considered.

As with grade 2 DIILD, the prognosis of grade 3 depends primarily on the response to steroid therapy, the evolution of the clinical condition (daily patient’s assessment in the inpatient setting, and then monitoring by repeated HRCT scans and spirometry tests according to the indication of a specialist lung physician), the extent and type of the radiological picture (pulmonary fibrosis is unfavourable) and the degree of lung function impairment. The risk factors for a worse prognosis are the same as for grade 2 DIILD (smoking history, type of drug, age and comorbidities, in particular those affecting the respiratory system).^41^

An illustrative grade 3 DIILD case study is reported in the Supplementary Materials, available at https://doi.org/10.1016/j.esmoop.2022.100404.

### Grade 4 DIILD

Grade 4 (very severe, life-threatening or disabling) DIILD is defined as the occurrence of severe, disabling symptoms leading to hospitalisation and possibly mechanical ventilatory support.^28^ The need for mechanical ventilatory assistance should take into account the patient’s baseline prognosis.

From an anatomopathological viewpoint, DAD is the dominant feature of grade 4 DIILD. DAD is characterised by an early (acute) exudative phase with oedema, the presence of hyaline membranes and inflammation, followed by an organising (subacute) phase with fibrosis, especially at the level of the alveolar septa, and hyperplasia of type II pneumocytes (Supplementary Figure S1E, available at https://doi.org/10.1016/j.esmoop.2022.100404). The presence of hyaline membranes is the pathognomonic sign of DAD.^64^ The disease can result in a complete *restitutio ad integrum* or it can progress to chronic parenchymal fibrosis.

**Figure 5:**
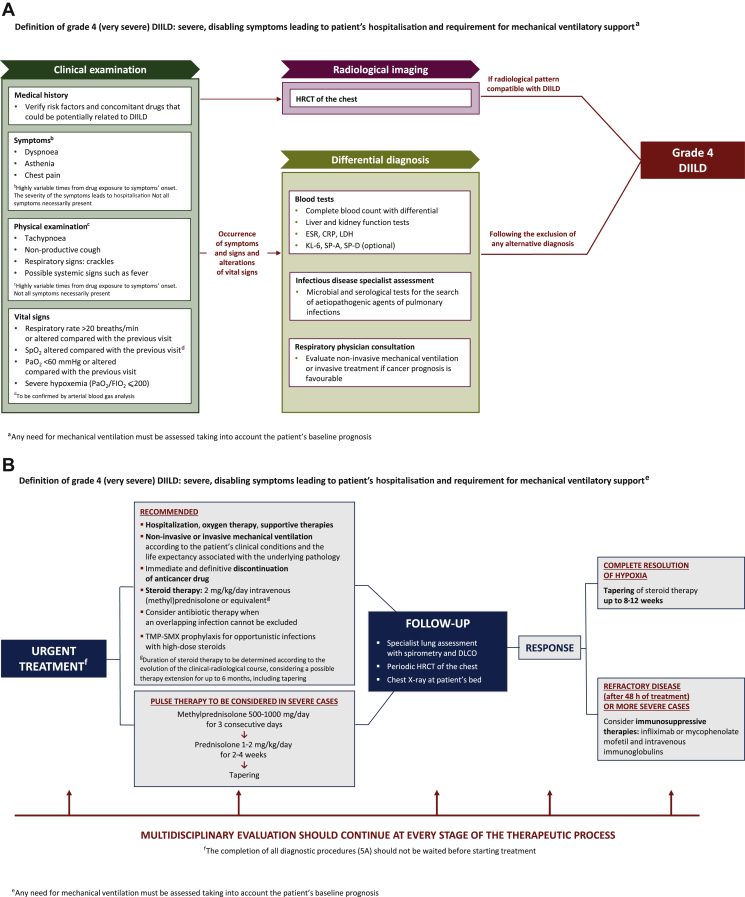
(A) Diagnostic algorithm and (B) algorithm for treatment and follow-up of grade 4 DIILD in cancer patients. Grade 4 DIILD definition is adapted from the Common Terminology Criteria for Adverse Events (CTCAE), Version 5.0 (27 November 2017).^28^ BAL, bronchoalveolar lavage; CRP, C-reactive protein; DIILD, drug-induced interstitial lung disease; DLCO, diffusing capacity of lung for carbon monoxide; ESR, erythrocyte sedimentation rate; FIO_2_, fraction of inspired oxygen; HRCT, high-resolution computed tomography; KL-6, Krebs von den Lungen-6; LDH, lactate dehydrogenase; PaO_2_, arterial partial pressure of oxygen; SpO_2_, oxygen saturation by pulse oximetry; TMP-SMX, trimethoprim/sulfamethoxazole.

A diagnostic algorithm for grade 4 DIILD in cancer patients receiving targeted therapy or immunotherapy is shown in Figure 5A. Patients with suspected grade 4 DIILD require an aggressive diagnostic work-up to rule out other pathologies such as infectious pneumonias or connective tissue diseases.^65^ Indeed, manifestations of grade 4 DIILD mimic those of ARDS, with acute onset and reduced oxygenation. The symptoms progress rapidly and, compared with the other DIILD grades, the clinical course is more abrupt.^66^ Patients with grade 4 DIILD typically manifest a significant hypoxaemia, with PaO_2_/FIO_2_ (fraction of inspired oxygen) ≤200, tachypnoea and the clinical features of ARDS.^67^

HRCT is highly sensitive in detecting pulmonary abnormalities and characterising lesions and allows, within certain limits, determination of a differential diagnosis.^68^ In the acute phase of grade 4 DIILD, the prevailing CT findings are diffuse and bilateral GGO, often with areas of lobular sparing which can be associated with local parenchymal consolidation or thickened interlobular septa with crazy-paving pattern. In the subacute phase, the radiological pattern is OP-like, with evidence of peribronchial or subpleural opacities. The reversed halo sign, although not pathognomonic, is a common CT finding in OP. Finally, in the chronic phase, there is fibrosis with irregular reticulation and traction bronchiectasis. The development of bronchiectasis is an ominous prognostic sign.^68^

A therapeutic algorithm for grade 4 DIILD in cancer patients receiving targeted therapy or immunotherapy is shown in Figure 5B. Urgent intervention is required with anticancer treatment interruption, supportive therapies, intravenous steroids, oxygen therapy and potentially mechanical ventilation. Non-invasive ventilation is preferred, but invasive mechanical ventilation may be considered in clinically severe cases, although the invasiveness of the intervention should be considered in the context of the patient’s cancer prognosis and aggressive manoeuvres avoided in patients with an unfavourable short-term prognosis. Mortality in patients with grade 4 DIILD is particularly high and often steroid therapy does not result in significant improvements. It generally seems that the efficacy of steroid therapy is higher in the OP pattern and, to a lesser extent, in the NSIP and HP patterns.^40^

Severe pneumonia is usually treated with intravenous (methyl)prednisolone 2 mg/kg/day. As an alternative for grade 4 DIILD, initial pulse therapy with methylprednisolone 500-1000 mg/day for 3 days should also be considered, followed by prednisolone 1-2 mg/kg/day for 2-4 weeks with subsequent tapering.^41^ Remission during steroid treatment does not confirm the diagnosis of DIILD, as other non-infectious interstitial pneumonias also respond to this therapy. In patients who show a good response, the dose of steroids should be progressively reduced over 8-12 weeks. Early interruption of steroid treatment or an excessively fast tapering may reactivate the disease. For patients who are steroid-refractory (e.g. no clinical improvement after 48 h) or have particularly severe DIILD, treatment with infliximab, mycophenolate mofetil or intravenous immunoglobulins should be considered.^7^

An early diagnosis of grade 4 DIILD is essential for a better prognosis, as this increases the likelihood of achieving a complete remission. Prognosis varies depending on the causative drug and the radiological pattern, with mortality rates of nearly 60%.^69^^,^^70^ From a radiological standpoint, the increased risk of mortality is associated with a DAD pattern, honeycombing and a diffuse and homogeneous pulmonary involvement. Finally, male sex, age >65 years, pre-existing lung disease and a diagnosis of non-small-cell lung cancer are commonly considered as risk factors for a worse prognosis,^41^ although further studies are needed to better identify patients at greatest risk of grade 4 DIILD.

An illustrative grade 4 DIILD case study is reported in the Supplementary Materials, available at https://doi.org/10.1016/j.esmoop.2022.100404.

## Conclusion

Hitherto, no consensus or standardised guidelines have been available to guide clinicians in the diagnostic work-up and optimal treatment of DIILD specifically in cancer patients. The aim of this expert opinion is to raise awareness for DIILD management, by providing a step-by-step diagnostic and therapeutic procedure for each grade of DIILD. Indeed, the number of targeted and immunological agents potentially associated with DIILD and now available in the therapeutic armamentarium for cancer is constantly growing. As a consequence, the caseload of DIILD associated with these agents may be expanding considerably in the real-world setting.

Clinical experience has demonstrated that, although potentially serious and life-threatening, DIILD is treatable if timely and accurately diagnosed, early and appropriately managed and strictly monitored. Effective management of DIILD in the oncology setting is built upon multidisciplinary interaction between oncologists, radiologists, pneumologists, pharmacologists and infectious disease specialists in all procedural phases, and on early detection and immediate intervention. Furthermore, increasing patients’ education can allow to ensure they pay close attention to their symptoms and report any changes to their physician/supportive care group immediately. Further improvements can be achieved through research on the underlying mechanisms of DIILD, diagnostic methodologies (e.g. identification of reliable molecular biomarkers) and effective therapeutic strategies.
