# Supplementary material for: Drug-induced interstitial lung disease during cancer therapies: expert opinion on diagnosis and treatment
Source: ESMO Open. 2022 Feb 24;7(2):100404. doi: 10.1016/j.esmoop.2022.100404 (PMC8881716; doi:10.1016/j.esmoop.2022.100404)
Supplement: Supplemental Material [file mmc1.docx]

**Drug-induced interstitial lung disease during cancer therapies: expert opinion on diagnosis and treatment**

**Supplemental Material**

**Tables**

Table S1. Classes of anticancer agents associated with DIILD in cancer patients and related incidence.

| **Class** | **Estimated DIILD incidence (range)^a^** |
| --- | --- |
| Bleomycin^1^ | 7%-21% |
| EGFR inhibitors^1,2^ | 0.3%-6% |
| HER2 inhibitors (including ADCs)^3-5^ | 2.4% (up to 15.8% with T-DXd) |
| BRC/ABL tyrosine kinase inhibitors^6-8^ | Unknown (imatinib and dasatinib); ≥1/1,000 to <1/100 (nilotinib) |
| ALK inhibitors^9^ | 2.5% (95% CI: 1.7%-3.6%) |
| BRAF inhibitors^10^ | 2.4% (trametinib); data not shown |
| PI3K inhibitors^11^ | 3%-25% |
| FLT3 inhibitors^12^ | 7%-8.5% |
| TRK/ROS1 inhibitors^13^ | Data not shown |
| VEGFR inhibitors^14^ | 0.37% (bevacizumab in combination with FOLFOX and FOLFIRI) |
| Immune checkpoint inhibitors^1^ | 1.1%-3.6% |
| CDK4/6 inhibitors^15^ | 0.3%-2.1% |
| mTOR inhibitors^16^ | 11% |
| PARP inhibitors^17,18^ | 0.79%-2.0% |

^a^Based on meta-analyses, systematic reviews and summaries of product characteristics (not unanimous diagnostic criteria and types of studies)

ADC, antibody-drug conjugate; ADP, adenosine diphosphate; ALK, anaplastic lymphoma kinase; CDK, cyclin-dependent kinase; CI, confidence interval; DIILD, drug-induced interstitial lung disease; EGFR, epidermal growth factor receptor; FLT3, FMS-like tyrosine kinase 3; FOLFIRI, folinic acid, 5-fluorouracil, irinotecan; FOLFOX, folinic acid, 5-fluorouracil, oxaliplatin; HER2, human epidermal growth factor receptor 2; mTOR, mammalian target of rapamycin; PARP, poly (ADP-ribose) polymerase; PI3K, phosphoinositide 3-kinase; T-DXd, trastuzumab-deruxtecan; VEGFR, vascular endothelial growth factor receptor

**Table S2.** Risk factors for DIILD in cancer patients.^1,19,20^

| **Patient-related risk factors** |
| --- |
| ***Major risk factors:*** |
| - History of cigarette smoking (≥50 packs/year) |
| - Pre-existing lung disease (such as IPF, COPD and bronchiectasis) |
| - Previous DIILD |
| - ECOG PS ≥2 at cancer diagnosis |
| - Advanced stage of the underlying cancer |
| ***Other risk factors:*** |
| - Increased age |
| - Male gender |
| - Asian ethnicity |
| - Prior thoracic radiotherapy |
| - Prior chemotherapy |
| - Underlying pulmonary neoplasm |
| - Comorbidities (e.g. kidney failure, diabetes) |
| - Genetic predisposition/Family history (e.g. cytochrome P450 enzyme polymorphisms and HLA allelic variants) |
| - Alcohol consumption |
| **Cancer treatment-related risk factors** |
| - Cumulative dose (bleomycin) and treatment duration^a^ |
| - Combinations of anticancer drugs |
| - Interaction with concomitant non-anticancer drugs |

^a^To be defined according to the drug involved (bleomycin, immune checkpoint inhibitors, tyrosine kinase inhibitors)

COPD, chronic obstructive pulmonary disease; DIILD, drug-induced interstitial lung disease; ECOG PS, Eastern Cooperative Oncology Group Performance Status; HLA, human leukocyte antigen; IPF, idiopathic pulmonary fibrosis

**Table S3.** Non-anticancer drugs associated with DIILD and related incidence.

| **Non-anticancer drugs** | **Estimated DIILD incidence (range)^a^** |
| --- | --- |
| Acetylsalicylic acid^21^ | Between 4% (adult general population) and 25% (asthma patients) |
| Amphotericin B^22^ | Data not shown |
| Amiodarone^1^ | 1.2%-8.8% |
| Azathioprine^23^ | >1/10,000, <1/1,000 |
| Beta blockers^24^ | Data not shown |
| Carbamazepine^25^ | <1/10,000 |
| Clarithromycin^26^ | Data not shown |
| Diclofenac^27^ | <1/10,000 |
| Granulocyte colony stimulating factor^1^ | 0.2% |
| Phenytoin^28^ | Data not shown |
| Fluoxetine^29^ | ≥1/10,000, <1/1,000 |
| Hydralazine^30^ | Data not shown |
| Levofloxacin^31^ | Data not shown |
| Contrast media^32^ | Unknown |
| Minocycline^33^ | ≥1/10,000, <1/1,000 |
| Naproxen^34^ | Rare |
| Nitrofurantoin^1^ | 3.65% |
| Gold^35^ | Cases reported |
| Paracetamol^36^ | Data not shown |
| Penicillamine | Data not shown |
| Penicillins^37^ | Data not shown |
| Statins^1^ | 1/40 of the AEs reported for statins |
| Sulfasalazine^38^ | Unknown |

^a^Based on meta-analyses, systematic reviews and summaries of product characteristics (not unanimous diagnostic criteria and type of studies)

AE, adverse event; DIILD, drug-induced interstitial lung disease

**Table S4.** DIILD classification according to CTCAE v5.0 (Adapted from the definition of pneumonia in the CTCAE v5.0).^39^

| **Grade** | **Clinical severity** |
| --- | --- |
| Grade 1 (mild) | Asymptomatic patient with radiographic findings only |
| Grade 2 (moderate) | Mild respiratory symptoms that do not deteriorate the patient’s quality of life |
| Grade 3 (severe) | Symptoms that lead to a worsening of the quality of life and limit the activities of daily living of the patient, possibly needing oxygen therapy, regardless of the severity of the radiologic findings |
| Grade 4 (very severe, life-threatening or disabling) | Severe, disabling symptoms leading to patient’s hospitalization and requirement for mechanical ventilatory support |
| Grade 5 (fatal) |  |

CTCAE, Common Terminology Criteria for Adverse Events; DIILD, drug-induced interstitial lung disease

**Table S5.** Infectious agents responsible for ILD.

| **Virus** | **Bacteria** | **Fungi** | **Protozoa** |
| --- | --- | --- | --- |
| Influenza viruses (A and B)  Respiratory syncytial virus (RSV)  Severe acute respiratory syndrome coronavirus 2  (SARS-CoV-2)  Other coronaviruses  Metapneumovirus  Parainfluenza virus  Rhinovirus  Adeno/enterovirus  Cytomegalovirus (CMV)  Epstein-Barr virus (EBV)  Herpes simplex virus (HSV) 1, 2, 7, 8 | *Mycoplasma pneumoniae*  *Chlamydia pneumoniae*  *Chlamydia psittaci*  *Coxiella burnetii*  *Mycobacterium tuberculosis*  *Mycobacterium avium complex* (MAC)  *Legionella pneumophila* | *Pneumocystis jirovecii*  *Aspergillus*  *Cryptococcus*  Endemic mycoses | *Toxoplasma* |

ILD, interstitial lung disease

**Table S6.** Dose adjustment recommendations for anticancer drugs at higher risk for DIILD.

| **Agent** | **Severity** | **Dose adjustment recommendations** |
| --- | --- | --- |
| Everolimus^16,40^ | Grade 1 | Evaluate continuation if no other relevant risk factors for severe DIILD are present |
|  | Grade 2 | Consider interruption of therapy until symptoms improve to grade 1. Reinitiate treatment at 5 mg daily. Discontinue treatment if failure to recover within 4 weeks |
|  | Grade 3 | Interrupt treatment until symptoms resolve to grade 1. Consider reinitiating treatment at 5 mg daily. If toxicity recurs at grade 3, consider discontinuation |
|  | Grade 4 | Discontinue treatment |
| Osimertinib/gefitinib/erlotinib^41-44^ | Grade 1 | Consider interruption of therapy until full resolution. Drug rechallenge at the same dosage may then be suitable |
|  | Grade 2 | Interruption of therapy until full resolution. Then evaluation of drug rechallenge after careful consideration of the individual patient’s benefits and risk may be reasonable (possibly at half the dosage to test tolerability) |
|  | Grade 3-4 | Discontinue treatment |
| Trastuzumab-deruxtecan^45^ | Grade 1 | Interrupt until fully resolved, then:  - if resolved in 28 days or less from  date of onset, maintain dose;  - if resolved in greater than 28 days  from date of onset, reduce dose  one level |
|  | Grade 2-4 | Permanently discontinue treatment |
| Alectinib/crizotinib/ceritinib^46-48^ | Any grade | Immediately interrupt and permanently discontinue |
| Nivolumab/pembrolizumab/  atezolizumab/durvalumab/ ipilimumab^49,50^ | Grade 1 | Evaluate continuation if no other relevant risk factor for severe DIILD |
|  | Grade 2 | Withhold until full resolution, then rechallenge |
|  | Grade 3-4 | Permanently discontinue treatment |

DIILD, drug-induced interstitial lung disease

**Figures**

**Figure S1. (A)** Computed tomography (CT) signs of acute interstitial pneumonia (AIP): hazy ground glass areas distributed bilaterally in the upper lobes (arrows); **(B)** CT signs of organizing pneumonia (OP): parenchymal consolidation in the right upper lobe posterior segment (arrow) with signs of centrilobular and paraseptal emphysema; **(C)** CT signs of nonspecific interstitial pneumonia (NSIP): mild traction bronchiectasis (arrows) and hazy ground glass areoles in the right lower lobe (asterisk); **(D)** CT signs of hypersensitivity pneumonia (HP): centrilobular micronodules in the right upper lobe posterior segment (arrows); (**E**) Diffuse alveolar damage (DAD) caused by immune checkpoint inhibitors.


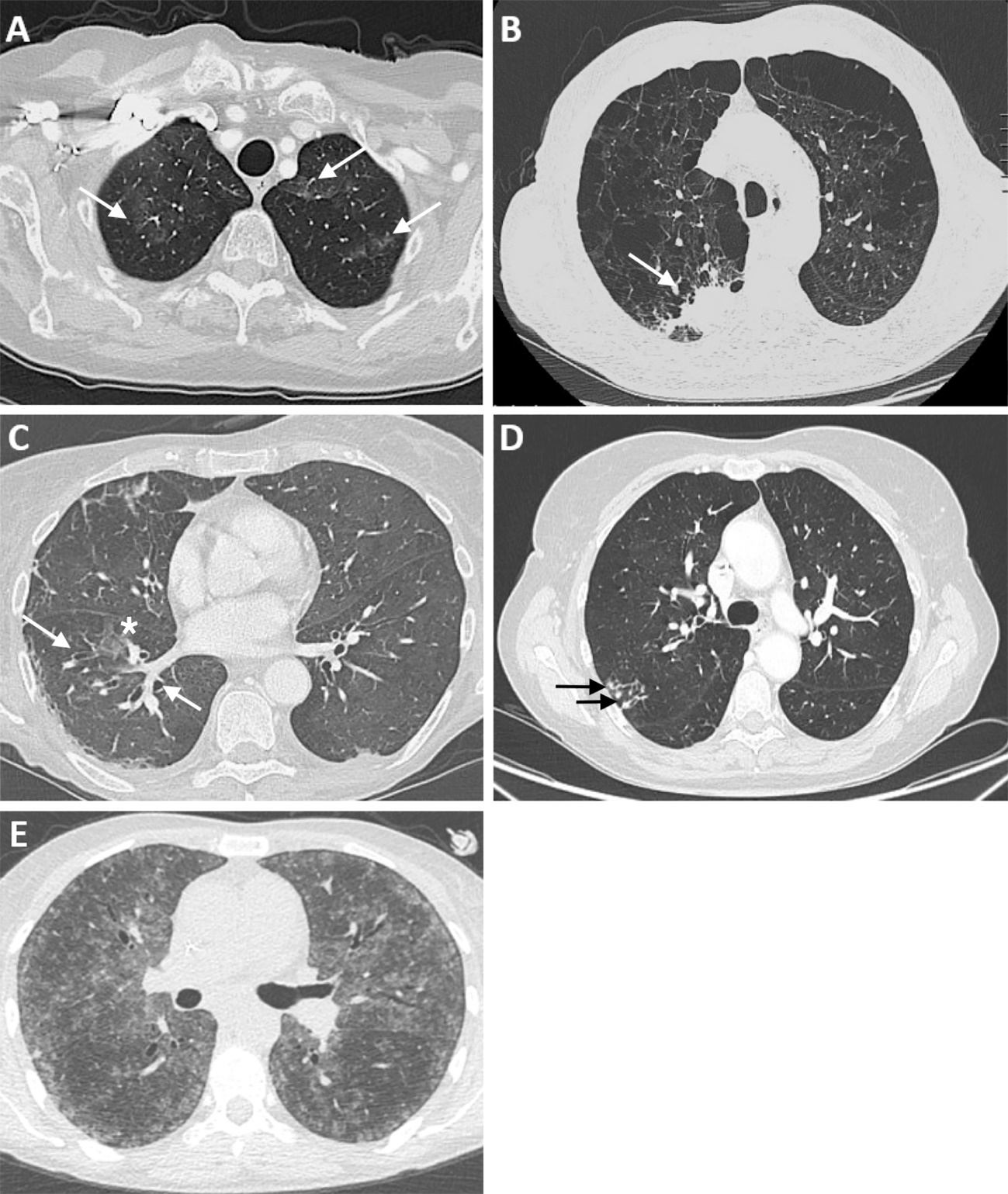


**Figure S2.** **(A)** Patient with *Legionella* pneumonia: ground glass opacities (GGOs) in the left lung with septal thickening and parenchymal consolidation in the right lung; **(B)** H1N1 influenza-related pneumonia: GGO in central and peripheral distribution in the right upper lobe; **(C)** Coronavirus disease 19 (COVID-19) viral pneumonia with multifocal GGO areas in the left upper lobe; **(D)** COVID-19 viral pneumonia: crazy-paving pattern in right and left lower lobes; **(E-F)** Metastatic lesion of the left lower lobe pre- (**E**) (arrow) and post- (**F**) stereotactic radiotherapy.


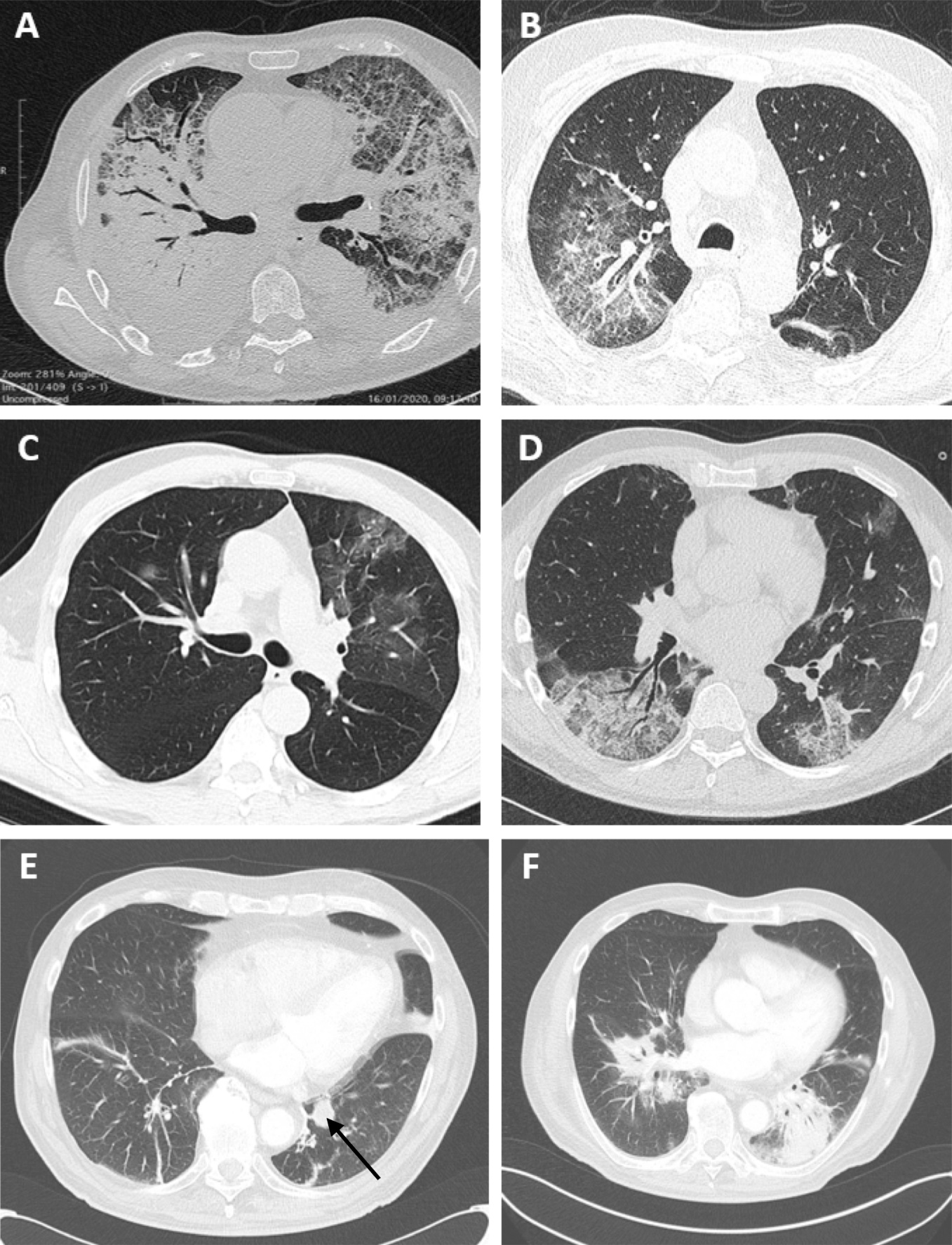


**Illustrative case reports**

**Grade 1 DIILD case study**

A 61-year-old woman with metastatic melanoma was treated with a combination therapy of immune checkpoint inhibitors (ICIs) and was diagnosed with asymptomatic grade 1 drug-induced interstitial lung disease (DIILD) with computed tomography (CT) signs of cryptogenic organising pneumonia (OP) (**Figure 1**). The discontinuation of anticancer therapy and the administration of prednisone 75 mg (then tapering off) led to DIILD remission.

**Figure 1.** Patches of parenchymal thickening as inflammation in the lower lobes (black arrows) and in the lingular segment (arrowheads).


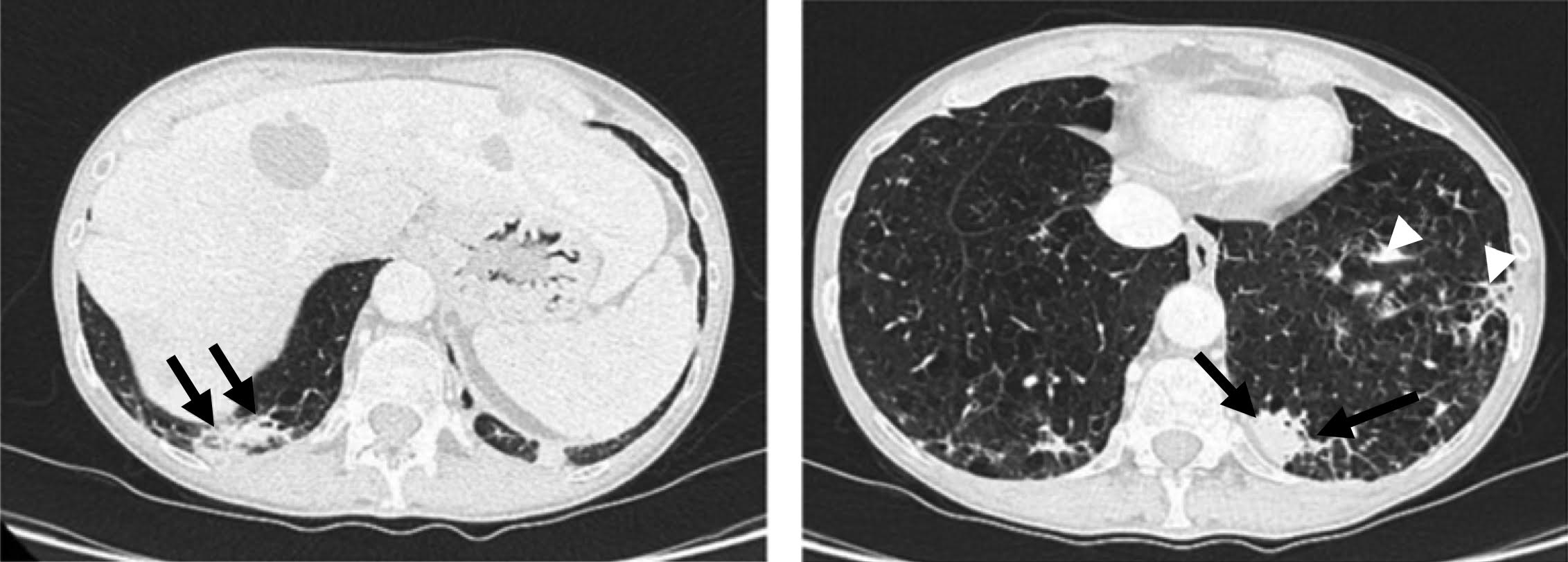


**Grade 2 DIILD case study**

A 62-year-old female with non-small cell lung cancer was being treated with ICI therapy and presented with fever, dyspnoea and cough related to pneumonia, without evidence of acute respiratory failure (arterial partial pressure of oxygen [PaO_2_] >60 mmHg). She was diagnosed with grade 2 DIILD with CT signs of acute interstitial pneumonia (AIP) (**Figure 2**).

**Figure 2**. **(A, B)** Diffuse bilateral ground glass opacity in the upper lobes (arrows); concomitant heteroplastic lesion in the left upper lobe apicoposterior segment; **(C)** Radiological remission following appropriate therapy (prednisone, levofloxacin and ceftriaxone).


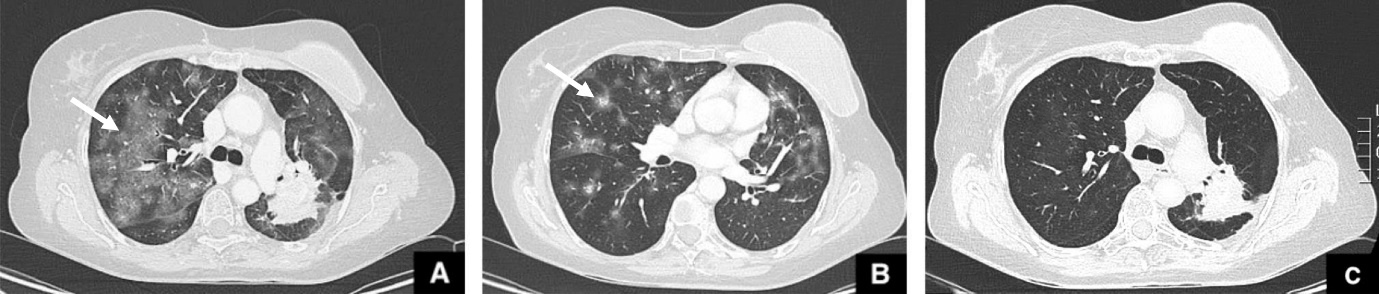


**Grade 3 DIILD case study**

A 68-year old woman was receiving ICI therapy for metastatic adenocarcinoma of the colon with microsatellite instability. She presented with cough, fever, and dyspnoea secondary to acute respiratory failure. A nasopharyngeal swab for SARS-CoV-2 was negative. She was diagnosed with grade 3 DIILD with CT signs of cryptogenic OP (**Figure 3**).

**Figure 3. (A)** Bilateral diffuse consolidation in the lower lobes, with peripheral distribution (arrows); **(B)** Regression of the radiological features after treatment with steroid therapy.


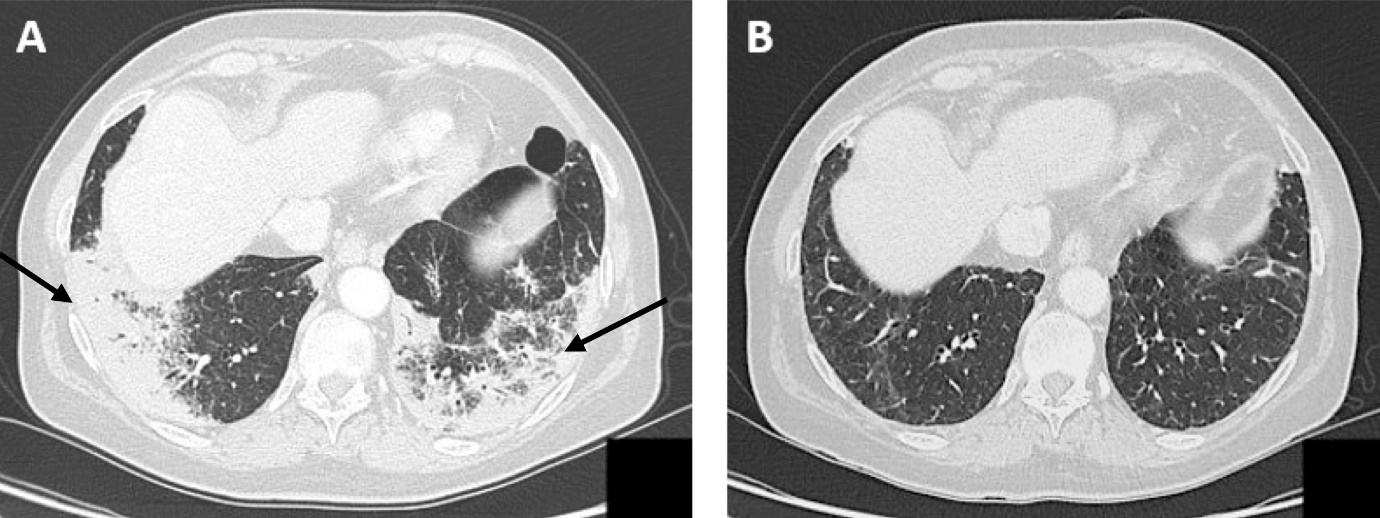


**Grade 4 DIILD case study**

A 73-year-old male with melanoma of the right scapula undergoing exeresis (pT1N2M0 stage IIIC, 2 local lymph nodes, *BRAF* wild-type) was treated with ICI therapy. One month after starting treatment, a positron emission tomography scan showed multiple bilateral hypermetabolic areas, likely inflammatory. The patient developed fever, with SpO_2_ (oxygen saturation by pulse oximetry) of 93%, one week after the scan. A SARS-CoV-2 swab was negative. OP-like pattern was found on CT (Figure 4A). After 3 days of antibiotic therapy, his symptoms had progressively worsened (dyspnoea, persistent fever at 38°C, respiratory failure not manageable with oxygen alone), and he had bilateral basal crackles. He was diagnosed with grade 4 DIILD and transferred to the intensive care unit, where he received continuous positive airway pressure therapy, methylprednisolone 80 mg/day and high-dose immunoglobulin therapy. After 2 weeks, the follow-up CT showed areas of parenchymal consolidation, extensive crazy-paving pattern and distortion of the lung structure (Figure 4B). The patient gradually improved although with persistent acquired pulmonary function impairment.

**Figure 4.** **(A)** Bilateral ground glass-like thickening in both lower lobes; **(B)** Areas of parenchymal consolidation, extensive crazy-paving pattern and distortion of the lung structure.


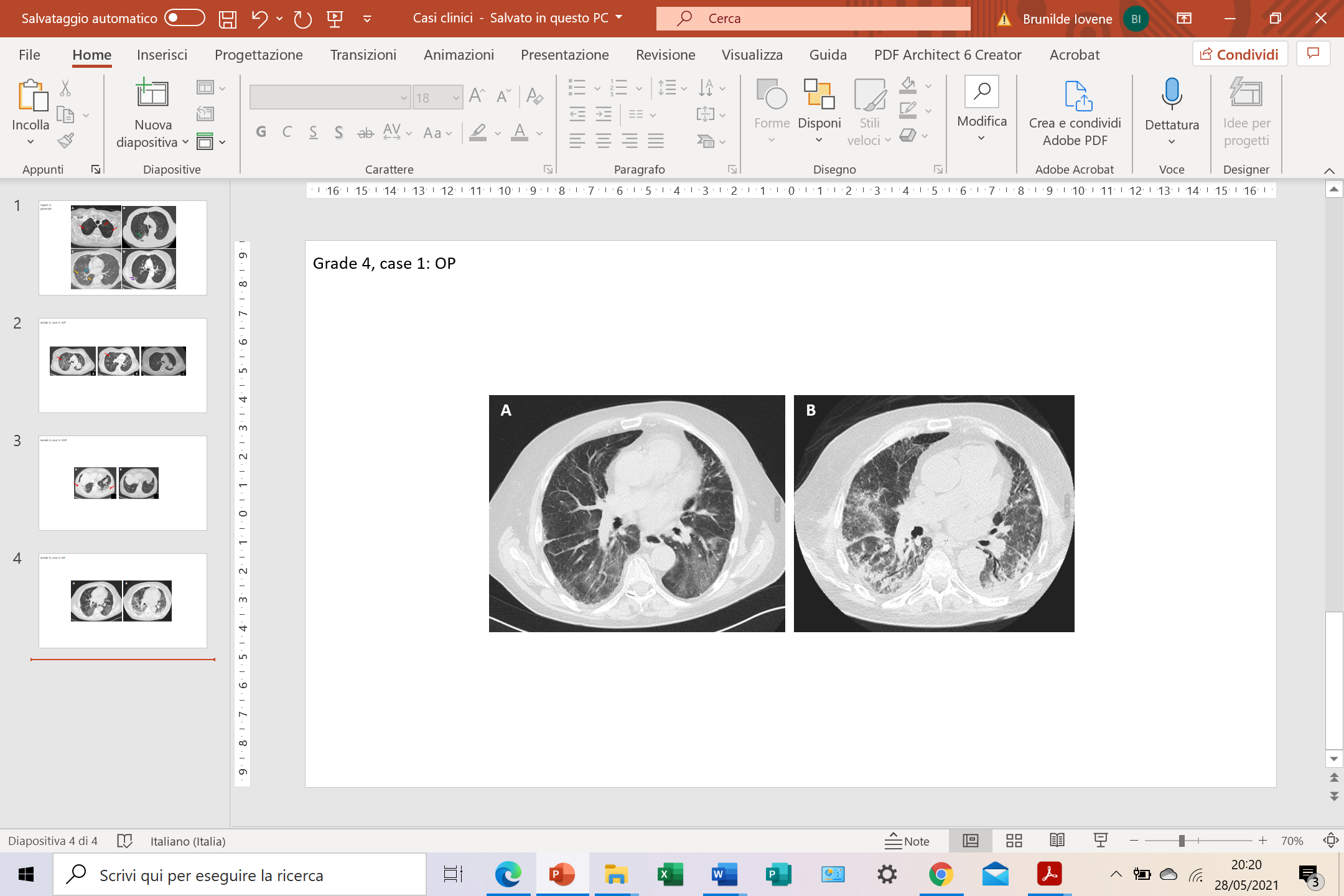


**References**

1. Skeoch S, Weatherley N, Swift AJ, et al. Drug-induced interstitial lung disease: a systematic review. *J Clin Med.* 2018;7(10).

2. Chang HL, Chen YH, Taiwan HC, Yang CJ. EGFR tyrosine kinase inhibitor-associated interstitial lung disease during the coronavirus disease 2019 pandemic. *J Thorac Oncol.* 2020;15(8):e129-e131.

3. Hackshaw MD, Danysh HE, Singh J, et al. Incidence of pneumonitis/interstitial lung disease induced by HER2-targeting therapy for HER2-positive metastatic breast cancer. *Breast Cancer Res Treat.* 2020 Aug;183(1):23-39.

4. Barroso-Sousa R, Tolaney SM. Clinical development of new antibody-drug conjugates in breast cancer: to infinity and beyond. *BioDrugs.* 2021 Mar 5:1-16.

5. Powell CA, Modi S, Iwata H, et al. Pooled analysis of drug-related interstitial lung disease (ILD) in 8 single-arm trastuzumab deruxtecan (T-DXd) studies [abstract]. In: Proceedings of the American Association for Cancer Research Annual Meeting 2021; 2021 Apr 10-15 and May 17-21. Philadelphia (PA): AACR; Cancer Res. 2021;81(13_Suppl):Abstract nr CT167.)

6. European Medicines Agency. Glivec (imatinib). Summary of Product Characteristics. Available at <https://www.ema.europa.eu/en/documents/product-information/glivec-epar-product-information_en.pdf>. Published 2021. Updated 12 May 2021. Accessed 24 October, 2020.

7. European Medicines Agency. Sprycel (dasatinib). Summary of Product Characteristics. Available at <https://www.ema.europa.eu/en/documents/product-information/sprycel-epar-product-information_en.pdf>. Published 2021. Updated 23 June 2021. Accessed 24 October, 2020.

8. European Medicines Agency. Tasigna (nilotinib). Summary of Product Characteristics. Available at <https://www.ema.europa.eu/en/documents/product-information/tasigna-epar-product-information_en.pdf>. Published 2021. Updated 21 July 2021. Accessed 24 October, 2020.

9. Lin L, Zhao J, Kong N, et al. Meta-analysis of the incidence and risks of interstitial lung disease and QTc prolongation in non-small-cell lung cancer patients treated with ALK inhibitors. *Oncotarget.* 2017;8(34):57379-57385.

10. Welsh SJ, Corrie PG. Management of BRAF and MEK inhibitor toxicities in patients with metastatic melanoma. *Ther Adv Med Oncol.* 2015;7(2):122-136.

11. Curigliano G, Shah RR. Safety and tolerability of phosphatidylinositol-3-kinase (PI3K) inhibitors in oncology. *Drug Saf.* 2019;42(2):247-262.

12. European Medicines Agency. Rydapt (midostaurin). Summary of Product Characteristics. Available at <https://www.ema.europa.eu/en/documents/product-information/rydapt-epar-product-information_en.pdf>. Published 2021. Updated 1 February 2021. Accessed 24 October, 2020.

13. European Medicines Agency. Rozlytrek (entrectinib). Summary of Product Characteristics. Available at <https://www.ema.europa.eu/en/documents/product-information/rozlytrek-epar-product-information_en.pdf>. Published 2020. Updated 27 October 2020. Accessed 24 October, 2020.

14. Saito Y, Gemma A. Current status of DILD in molecular targeted therapies. *Int J Clin Oncol.* 2012;17(6):534-541.

15. Raschi E, Fusaroli M, Ardizzoni A, et al. Cyclin-dependent kinase 4/6 inhibitors and interstitial lung disease in the FDA adverse event reporting system: a pharmacovigilance assessment. *Breast Cancer Res Treat.* 2021;186(1):219-227.

16. Willemsen AE, Grutters JC, Gerritsen WR, van Erp NP, van Herpen CM, Tol J. mTOR inhibitor-induced interstitial lung disease in cancer patients: comprehensive review and a practical management algorithm. *Int J Cancer.* 2016;138(10):2312-2321.

17. Ma Z, Sun X, Zhao Z, et al. Risk of pneumonitis in cancer patients treated with PARP inhibitors: a meta-analysis of randomized controlled trials and a pharmacovigilance study of the FAERS database. *Gynecol Oncol.* 2021 Aug;162(2):496-505.

18. Poveda A, Floquet A, Ledermann JA, et al; SOLO2/ENGOT-Ov21 investigators. Olaparib tablets as maintenance therapy in patients with platinum-sensitive relapsed ovarian cancer and a BRCA1/2 mutation (SOLO2/ENGOT-Ov21): a final analysis of a double-blind, randomised, placebo-controlled, phase 3 trial. *Lancet Oncol.* 2021 May;22(5):620-631.

19. Ratwani A, Gupta A, Stephenson BW, et al. The spectrum of drug-induced interstitial lung disease. *Current Pulmonology Reports.* 2019;8:139-150.

20. Schwaiblmair M, Behr W, Haeckel T, et al. Drug induced interstitial lung disease. *Open Respir Med J.* 2012;6:63-74.

21. Distefano G, Fanzone L, Palermo M, et al. HRCT patterns of drug-induced interstitial lung diseases: a review. *Diagnostics (Basel).* 2020;10(4).

22. AmBisome (liposomal amphotericin B). Summary of Product Characteristics. Available at <https://www.hpra.ie/img/uploaded/swedocuments/Licence_PA2322-001-001_22112018145008.pdf>. Published 2018. Accessed 24 October, 2020.

23. Azafor (azathioprine). Summary of Product Characteristics. Available at <https://mri.cts-mrp.eu/Human/Downloads/IT_H_0273_001_FinalSPC.pdf>. Published 2012. Accessed 24 October, 2020.

24. Nebilet (nebivolol). Summary of Product Characteristics. Available at <https://www.hpra.ie/img/uploaded/swedocuments/Licence_PA0865-015-002_19022021154301.pdf>. Published 2021. Updated 19 February 2021. Accessed 24 October, 2020.

25. Carbamazepine. Summary of Product Characteristics. Available at <https://mri.cts-mrp.eu/human/downloads/NL_H_0238_003_FinalLabelling_3of4.pdf>. Published 2014. Accessed 24 October, 2020.

26. Claritromicina Pensa. Summary of Product Characteristics. Available at <https://www.codifa.it/farmaci/c/claritromicina-pensa-claritromicina-antibatterici-macrolidi>. Published 2021. Updated 20 July 2021. Accessed 24 October, 2020.

27. Diclofenac sodium. Summary of Product Characteristics. Available at <http://mri.cts-mrp.eu/download/NL_H_0173_004_FinalPI_1of2.pdf>. Published 2021. Accessed 24 October, 2020.

28. Phenytoin (Hikma) solution for injection. Summary of Product Characteristics. Available at <https://farmaci.agenziafarmaco.gov.it/aifa/servlet/PdfDownloadServlet?pdfFileName=footer_002653_038935_RCP.pdf&sys=m0b1l3>. Published 2010. Accessed 24 October, 2020.

29. Prozac (fluoxetine). Summary of Product Characteristics. Available at <https://mri.cts-mrp.eu/human/downloads/FR_H_0242_001_FinalPI.pdf>. Published 2021. Accessed 24 October, 2020.

30. Apresoline (hydralazine hydrochloride). Summary of Product Characteristics. Available at <https://www.hpra.ie/img/uploaded/swedocuments/LicenseSPC_PA1142-015-001_16022016090138.pdf>. Published 2016. Accessed 24 October, 2020.

31. Levofloxacin. Summary of Product Characteristics. Available at <https://mri.cts-mrp.eu/human/downloads/IT_H_0291_002_FinalSPC.pdf>. Published 2011. Accessed 24 October, 2020.

32. Iomeron. Summary of Product Characteristics. Available at <https://www.hpra.ie/img/uploaded/swedocuments/LicenseSPC_PA1826-006-004_19072018154101.pdf>. Published 2018. Accessed 24 October, 2020.

33. Minocycline. Summary of Product Characteristics. Available at <https://www.hpra.ie/img/uploaded/swedocuments/LicenseSPC_PPA1151-138-001_22102012165044.pdf>. Published 2012. Accessed 24 October, 2020.

34. Naproxen. Summary of Product Characteristics. Available at <https://mri.cts-mrp.eu/human/downloads/SE_H_2063_001_FinalSPC.pdf>. Published 2021. Accessed 24 October, 2020.

35. Ridaura. Summary of Product Characteristics. Available at <https://www.hpra.ie/img/uploaded/swedocuments/LicenseSPC_PA0899-011-001_03042009220031.pdf>. Published 2009. Accessed 24 October, 2020.

36. Paracetamol. Summary of Product Characteristics. Available at <https://www.accord-healthcare.com/ie/sites/default/files/spc/licence_pa2315-065-003_26092019151751.pdf>. Published 2019. Accessed 24 October, 2020.

37. Liderclox. Summary of Product Characteristics. Available at <https://www.codifa.it/farmaci/l/liderclox-flucloxacillina-sodica-antibatterici-penicillinici>. Published 2021. Updated 20 July 2021. Accessed 24 October, 2020.

38. Salazopyrin (sulfasalazine). Summary of Product Characteristics. Available at <https://www.hpra.ie/img/uploaded/swedocuments/Licence_PA0822-196-002_08112019140536.pdf>. Published 2019. Accessed 24 October, 2020.

39. US Department of Health and Human Services. Common Terminology Criteria for Adverse Events (CTCAE) Version 5.0. Available at <https://ctep.cancer.gov/protocoldevelopment/electronic_applications/docs/ctcae_v5_quick_reference_5x7.pdf>. Published 2017. Accessed 24 October, 2020.

40. European Medicines Agency. Afinitor (everolimus). Summary of Product Characteristics. Available at <https://www.ema.europa.eu/en/documents/product-information/afinitor-epar-product-information_en.pdf>. Published 2019. Accessed 31 December, 2021.

41. Kodama H, Wakuda K, Yabe M, et al. Retrospective analysis of osimertinib re-challenge after osimertinib-induced interstitial lung disease in patients with EGFR-mutant non-small cell lung carcinoma. Invest New Drugs. 2021 Apr;39(2):571-577.

42. Tagrisso (osimertinib). Summary of Product Characteristics. Available at <https://www.ema.europa.eu/en/documents/product-information/tagrisso-epar-product-information_en.pdf>. Published 2016. Accessed 31 December, 2021.

43. Iressa (gefitinib). Summary of Product Characteristics. Available at <https://www.ema.europa.eu/en/documents/product-information/iressa-epar-product-information_en.pdf>. Published 2014. Accessed 31 December, 2021.

44. Tarceva (erlotinib). Summary of Product Characteristics. Available at <https://www.ema.europa.eu/en/documents/product-information/tarceva-epar-product-information_en.pdf>. Published 2010. Accessed 31 December, 2021.

45. European Medicines Agency. Enhertu (trastuzumab deruxtecan). Summary of Product Characteristics. Available at <https://www.ema.europa.eu/en/documents/product-information/enhertu-epar-product-information_en.pdf>. Published 2021. Accessed 13 May, 2021.

46. European Medicines Agency. Alecensa (alectinib). Summary of Product Characteristics. Available at <https://www.ema.europa.eu/en/documents/product-information/alecensa-epar-product-information_en.pdf>. Published 2017. Accessed 31 December, 2021.

47. European Medicines Agency. Xalkori (crizotinib). Summary of Product Characteristics. Available at <https://www.ema.europa.eu/en/documents/product-information/xalkori-epar-product-information_en.pdf>. Published 2016. Accessed 31 December, 2021.

48. European Medicines Agency. Zykadia (ceritinib). Summary of Product Characteristics. Available at <https://www.ema.europa.eu/en/documents/product-information/zykadia-epar-product-information_en.pdf>. Published 2017. Accessed 31 December, 2021.

49. Haanen J, Carbonnel F, Robert C, et al. Management of toxicities from immunotherapy: ESMO clinical practice guidelines for diagnosis, treatment and follow-up. *Ann Oncol.* 2017;28(suppl_4):iv119-iv142.

50. Brahmer JR, Abu-Sbeih H, Ascierto PA, et al. Society for Immunotherapy of Cancer (SITC) clinical practice guideline on immune checkpoint inhibitor-related adverse events. *J Immunother Cancer.* 2021;9(6):e002435.
